# Oral administration of cannabis with lipids leads to high levels of cannabinoids in the intestinal lymphatic system and prominent immunomodulation

**DOI:** 10.1038/s41598-017-15026-z

**Published:** 2017-11-06

**Authors:** Atheer Zgair, Jong Bong Lee, Jonathan C. M. Wong, Dhiaa A. Taha, Jehan Aram, Daisy Di Virgilio, Joshua W. McArthur, Yu-Kit Cheng, Ivo M. Hennig, David A. Barrett, Peter M. Fischer, Cris S. Constantinescu, Pavel Gershkovich

**Affiliations:** 1 School of Pharmacy, University of Nottingham, Nottingham, NG7 2RD United Kingdom; 2 College of Pharmacy, University of Anbar, Anbar, Iraq; 3 Division of Clinical Neuroscience, University of Nottingham and Queen’s Medical Centre, Nottingham, NG7 2UH United Kingdom; 4 Nottingham City Hospital, Nottingham University Hospitals NHS Trust, Nottingham, NG5 1PB United Kingdom

## Abstract

Cannabidiol (CBD) and ∆^9^-tetrahydrocannabinol (THC) have well documented immunomodulatory effects *in vitro*, but not following oral administration in humans. Here we show that oral co-administration of cannabinoids with lipids can substantially increase their intestinal lymphatic transport in rats. CBD concentrations in the lymph were 250-fold higher than in plasma, while THC concentrations in the lymph were 100-fold higher than in plasma. Since cannabinoids are currently in clinical use for the treatment of spasticity in multiple sclerosis (MS) patients and to alleviate nausea and vomiting associated with chemotherapy in cancer patients, lymphocytes from those patients were used to assess the immunomodulatory effects of cannabinoids. The levels of cannabinoids recovered in the intestinal lymphatic system, but not in plasma, were substantially above the immunomodulatory threshold in murine and human lymphocytes. CBD showed higher immunosuppressive effects than THC. Moreover, immune cells from MS patients were more susceptible to the immunosuppressive effects of cannabinoids than those from healthy volunteers or cancer patients. Therefore, administering cannabinoids with a high-fat meal or in lipid-based formulations has the potential to be a therapeutic approach to improve the treatment of MS, or indeed other autoimmune disorders. However, intestinal lymphatic transport of cannabinoids in immunocompromised patients requires caution.

## Introduction

*Cannabis sativa* has a very long history of medical use. It contains unique biochemical compounds called cannabinoids of which cannabidiol (CBD) and ∆^9^-tetrahydrocannabinol (THC) are the most abundant. Both compounds have been extensively studied over the last few decades for multiple therapeutic effects with immunomodulatory effects recently receiving considerable attention^1,2^. CBD has been shown to be effective following oral administration in lipid-based formulations or parenteral injections in animal models of a number of debilitating diseases caused by over-reactive immune responses (autoimmune and inflammatory diseases) including multiple sclerosis (MS)^3^, rheumatoid arthritis^4^, diabetes mellitus^5^, allergic asthma^6^, autoimmune hepatitis^7^ and colitis^8^. Similarly, THC has been shown to be beneficial following oral administration in lipid-based formulations or parenteral injections in MS^9^, diabetes mellitus^10^, and allergic asthma^6^ animal models. The proposed mechanisms governing these effects involve the ability of CBD and THC at relatively high concentrations to suppress lymphocyte proliferation and inflammatory cytokine production^3–8,11^. This is supported by immunosuppressive effects that have been detected in *in vitro* studies^12,13^. Nevertheless, only a few human studies have been conducted to assess the immunomodulatory effects of cannabinoids in patients suffering from autoimmune diseases, particularly MS. These human studies showed no clear evidence for the immunosuppressive effects following oral administration of low-amount lipid-containing formulations of cannabinoids^14,15^. In fact, Killestein *et al*.^14^ suggested a moderate pro-inflammatory potential in MS patients treated with orally administered cannabis extract. Katona *et al*.^15^ showed that the discrepancy between animal and human studies could be, in part, due to the high oral doses of cannabinoids used in animals (8–40 mg/kg) compared to around 0.25 mg/kg in human trials. This results in plasma levels in humans of approximately 5 ng/mL on average^15^. These low levels of cannabinoids detected in human plasma are consistent with the extensive first-pass metabolism reported for CBD and THC after oral administration^16,17^.

Recently, we have demonstrated that oral co-administration of CBD and THC with sufficient amount of long-chain triglycerides (LCT), equivalent to a moderate- to high-fat meal in man, can enhance intestinal lymphatic transport and markedly increase systemic bioavailability of cannabinoids in rats^18^. In addition, high concentrations within the intestinal lymphatic system have previously been reported for compounds which are absorbed following oral administration into systemic circulation primarily through the intestinal lymphatic system^19–21^. Importantly, the intestinal lymphatic system is an essential contributor in the immune functions of the body^22^. It is the largest lymphatic organ and contains more than half of the body’s lymphocytes^23,24^. Therefore, the primary aim of our work was to assess whether the concentrations of CBD and THC found in the intestinal lymphatic system following oral co-administration with lipids could reach levels that are sufficiently high to produce immunomodulatory effects. MS and cancer patients on chemotherapy regimen were selected in this study as model cases of autoimmune illness and immunocompromised status, respectively, based on the fact that cannabinoids are currently used as symptomatic treatment in both patients groups^25,26^, and have immunomodulatory activity^1^. Therefore, an additional aim was to assess if such high levels in the intestinal lymphatic system are of potential therapeutic value to improve the treatment outcomes of autoimmune diseases such as MS, or can lead to potential safety considerations in immunocompromised patients such as those under chemotherapy regimens.

## Results

### Biodistribution of cannabinoids to rat mesenteric lymph nodes, spleen, lymph fluid and plasma following oral administration

**Figure 1:**
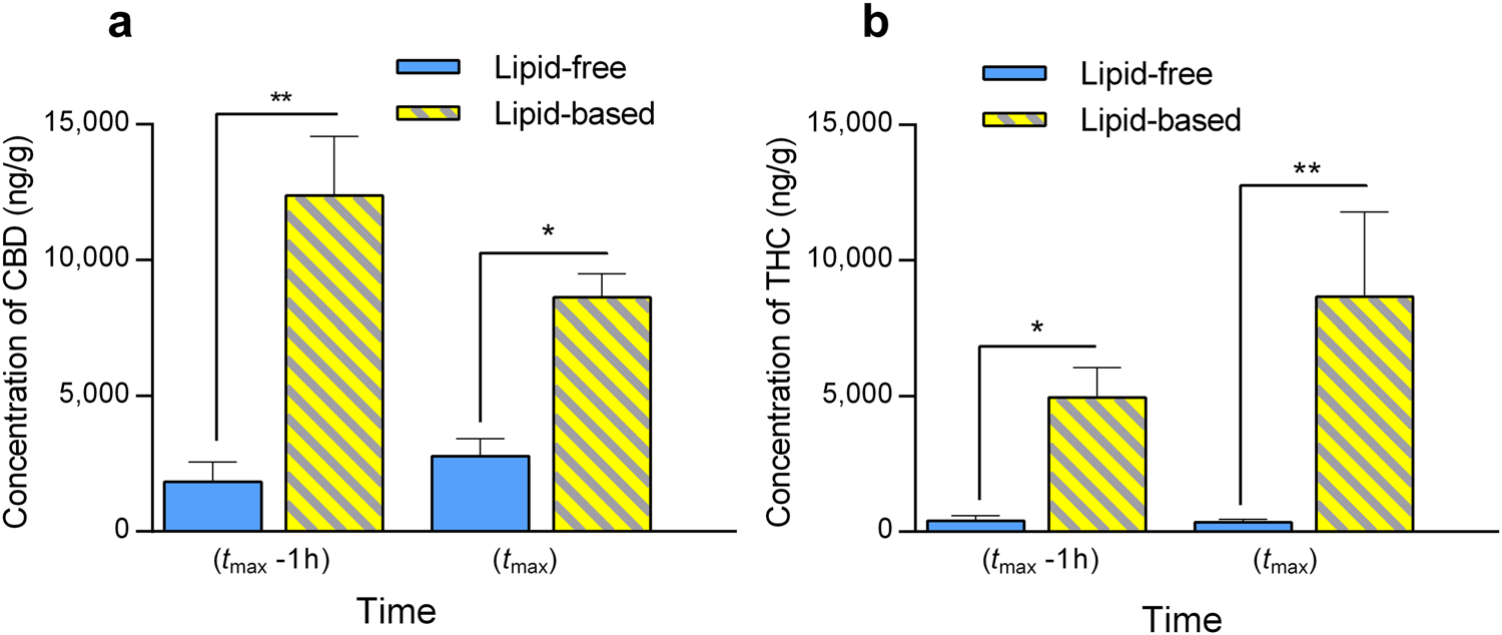
Distribution of cannabinoids to mesenteric lymph nodes (MLN). Cannabinoids were orally administered in lipid-free and lipid-based formulations (solution of cannabinoids in sesame oil) to rats. (**a**) Concentration of CBD recovered in MLN at time of maximum concentration in plasma (*t*_max_) and one-hour prior to *t*_max_ (*t*_max_ − 1 h) following oral administration of lipid-free formulation (12 mg/kg, *n* = 3 at *t*_max_, *n* = 3 at *t*_max_ − 1 h), and long-chain triglycerides (LCT)-based formulation (12 mg/kg, *n* = 3 at *t*_max_, *n* = 3 at *t*_max_ − 1 h) to rats. (**b**) Concentration of THC recovered in MLN at *t*_max_ and *t*_max_ − 1 h following oral administration of lipid-free formulation (12 mg/kg, *n* = 5 at *t*_max_, *n* = 4 at *t*_max_ − 1 h), and LCT-based formulation (12 mg/kg, *n* = 3 at *t*_max_, *n* = 4 at *t*_max_ − 1 h) to rats. Values are expressed as mean ± SEM. Statistical analysis was performed using unpaired two-tailed Student’s *t*-test. *P < 0.05; **P < 0.01.

**Figure 2:**
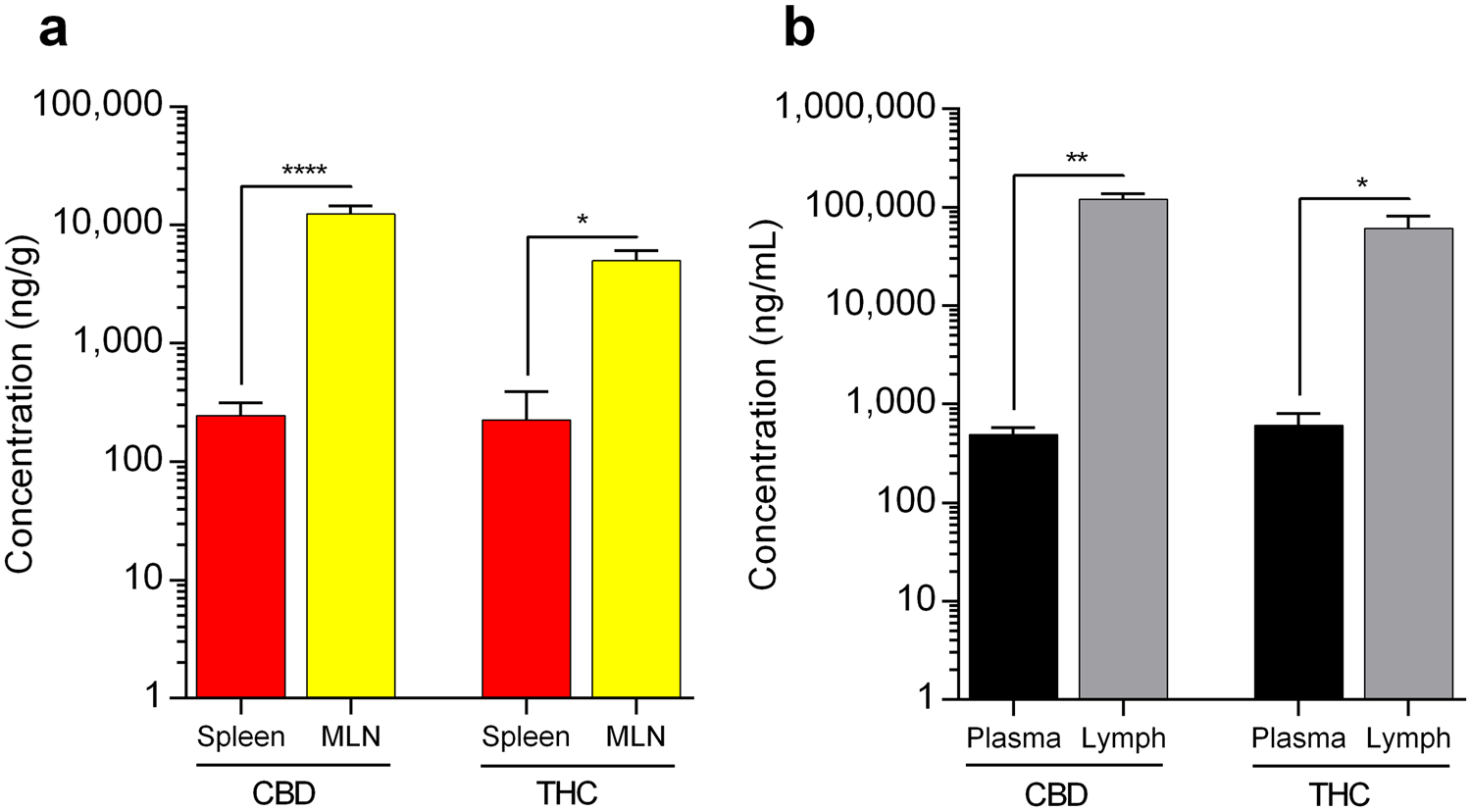
Concentration of cannabinoids recovered in spleen, mesenteric lymph nodes (MLN), plasma, and intestinal lymph fluid. Cannabinoids were orally administered in lipid-based formulations (solution of cannabinoids in sesame oil) to rats. (**a**) Concentration of CBD and THC in spleen (*n* = 6 and 3, respectively) and MLN (*n* = 3 and 4, respectively) two hours (one-hour prior to *t*_max_) following oral administration of long-chain triglyceride (LCT)-based formulation (12 mg/kg) to rats. (**b**) Concentration of CBD and THC in plasma (*n* = 3 and 4, respectively) and lymph (*n* = 3 and 4, respectively) two hours (one-hour prior to *t*_max_) following oral administration of LCT-based formulation (12 mg/kg) to rats. Values are expressed as mean ± SEM. Statistical analysis was performed using unpaired two-tailed Student’s *t*-test. *P < 0.05; **P < 0.01; ****P < 0.0001.

The biodistribution of CBD and THC to mesenteric lymph nodes (MLN) was assessed following oral administration in lipid-free and LCT-based (solution in sesame oil) formulations to rats at the time of maximum concentration in plasma (*t*_max_) and one-hour prior to *t*_max_. These time points were based on our earlier study of the plasma pharmacokinetics of CBD and THC in rats (Supplementary Table S1)^18^. The concentrations of CBD and THC recovered in MLN following oral administration with LCT were profoundly higher than those observed after administration with lipid-free formulation at both time points (Fig. 1a,b). To assess the effect of intestinal lymphatic transport on the exposure of lymphocytes to cannabinoids within the intestinal lymphatic system versus splenocytes, the concentrations of cannabinoids in MLN were compared with those found in spleen. Figure 2a shows that significantly higher levels of cannabinoids were found in MLN compared to spleen. The concentrations in MLN were more than 50-fold and 20-fold higher than in spleen for CBD and THC, respectively (Fig. 2a). Figure 2b shows the concentrations of CBD and THC in lymph fluid versus plasma. Profoundly higher concentrations of cannabinoids were observed in lymph fluid compared with plasma. CBD concentrations in intestinal lymph fluid were as much as 250-fold higher than the concentration in plasma, while the concentration of THC was 100-fold higher in lymph than in plasma.

### Effect of CBD and THC on the proliferation of immune cells isolated from MLN and spleen of rats

**Figure 3:**
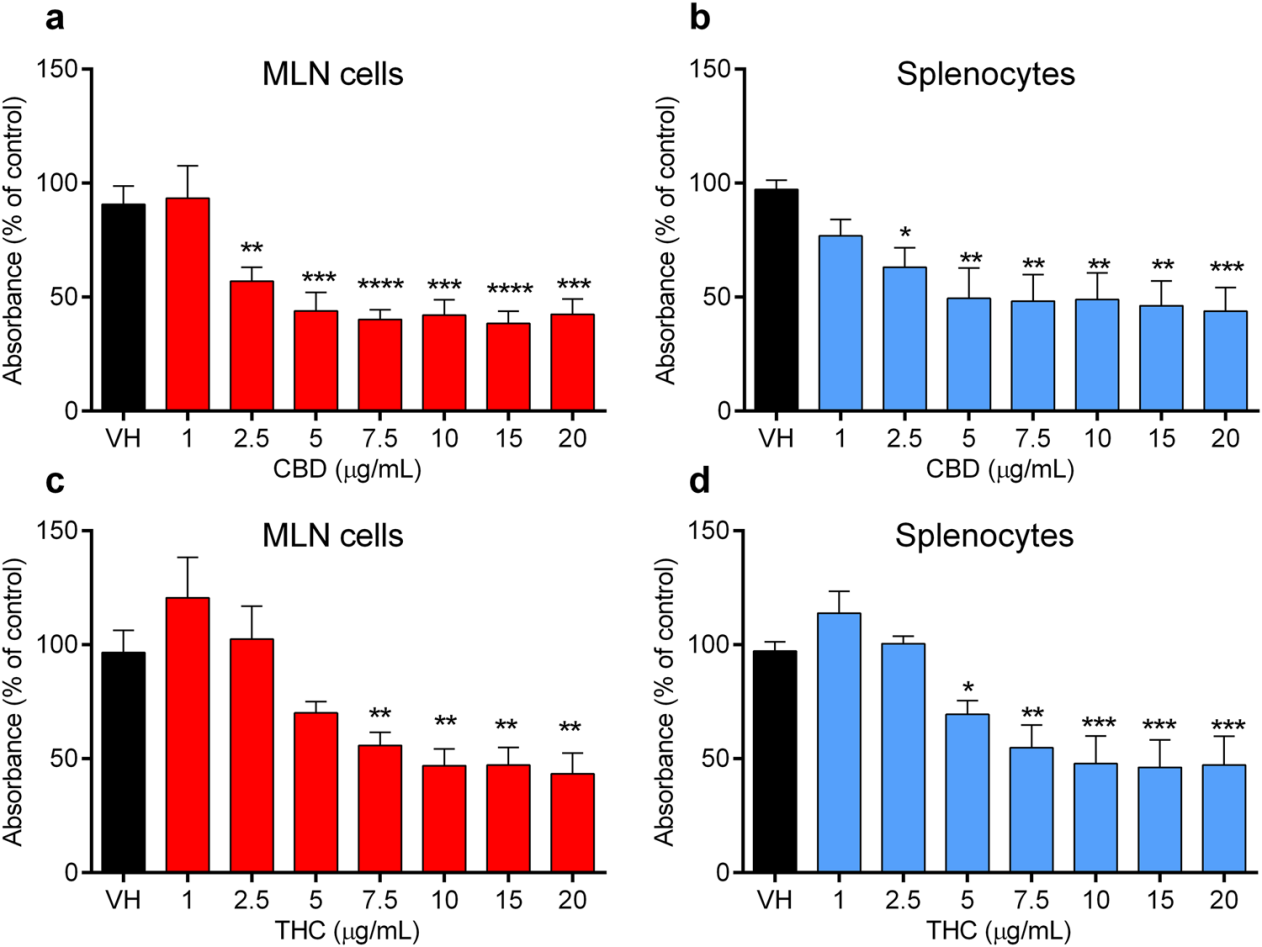
Effects of cannabinoids on the proliferation of immune cells isolated from mesenteric lymph nodes (MLN) and spleen of rats. Cells were stimulated by phytohaemagglutinin (PHA, 10 μg/mL) after incubation with CBD or THC at concentrations of 1–20 μg/mL. (**a**) Effect of CBD on the proliferation of immune cells isolated from MLN. (**b**) Effect of CBD on the proliferation of immune cells isolated from spleen. (**c**) Effect of THC on the proliferation of immune cells isolated from MLN. (**d**) Effect of THC on the proliferation of immune cells isolated from spleen. Values are expressed as percentage of absorbance compared to the control group of untreated cells, mean ± SEM (*n* = 5). Samples run in triplicates. Statistical analysis was performed using one-way ANOVA with Fisher’s LSD test. Statistical differences compared to the vehicle (DMSO)-treated cells (VH); *P < 0.05; **P < 0.01; ***P < 0.001; ****P < 0.0001.

Proliferation assays are commonly used to assess lymphocyte responses to a variety of stimuli^27^. We evaluated whether the concentration of cannabinoids achieved in the intestinal lymphatic system had immunomodulatory effects on immune cells isolated from MLN and the spleen of rats. CBD significantly suppressed mitogen-stimulated proliferation of immune cells from both MLN and spleen at concentrations equal to and above 2.5 μg/mL (Fig. 3a,b). Slightly higher concentrations of THC were required to inhibit the proliferation of immune cells isolated from MLN and spleen, at and above 7.5 and 5 μg/mL, respectively (Fig. 3c,d).

### CBD and THC attenuate the frequency of CD3^+^ T cells producing the pro-inflammatory cytokines, TNF-α and IFN-γ

**Figure 4:**
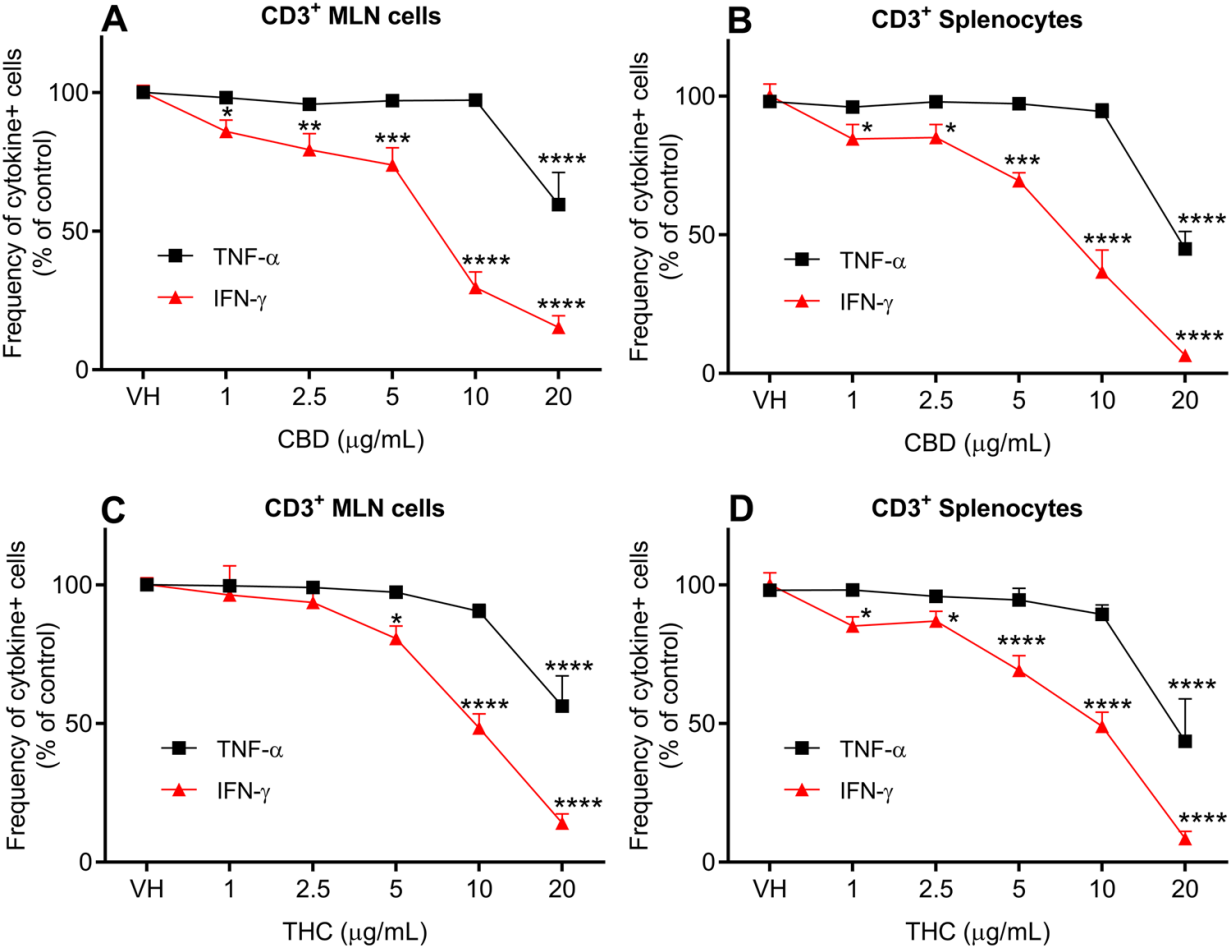
Effects of cannabinoids on the expression of inflammatory cytokines in immune cells isolated from rats. The figure shows the frequency of TNF-α and IFN-γ expressing CD3^+^ T cells isolated from mesenteric lymph nodes (MLN) and spleen of rats (% frequency of cytokines-expressing CD3^+^ T cells compared to the control group of untreated cells, mean ± SEM). Cells were stimulated by phorbol myristate acetate and ionomycin (PMA & I) in the presence of brefeldin A after incubation with CBD or THC at concentrations of 1–20 μg/mL. (**A**) Effect of CBD on the frequency of cytokines-expressing CD3^+^ T cells isolated from MLN (*n* = 7). (**B**) Effect of CBD on the frequency of cytokines-expressing CD3^+^ T cells isolated from spleen (*n* = 5). (**C**) Effect of THC on the frequency of cytokines-expressing CD3^+^ T cells isolated from MLN (*n* = 7). (**D**) Effect of THC on the frequency of cytokines-expressing CD3^+^ T cells isolated from spleen (*n* = 5). Statistical analysis was performed using one-way ANOVA with Fisher’s LSD test. Statistical differences compared to the vehicle (DMSO)-treated cells (VH); *P < 0.05; **P < 0.01; ***P < 0.001; ****P < 0.0001.

In addition to lymphocyte proliferation, the immunomodulatory effects of CBD and THC were assessed by measuring the intracellular expression of TNF-α and IFN-γ in CD3^+^ T cells isolated from MLN and spleen of rats. As shown in Fig. 4A,B, CBD significantly decreased TNF-α expressing T cells from both MLN and spleen only at relatively high concentrations (20 μg/mL). However, a more potent effect was observed for CBD on IFN-γ (1 µg/mL). Similar to CBD, THC also significantly reduced TNF-α expressing T cells from MLN and spleen at the highest tested concentration of 20 μg/mL. However, lower concentrations were required to significantly reduce IFN-γ expressing T cells from MLN and spleen, at 5 and 1 μg/mL, respectively (Fig. 4C,D).

### Effect of CBD and THC on the proliferation of peripheral blood mononuclear cells (PBMCs) isolated from human blood

**Figure 5:**
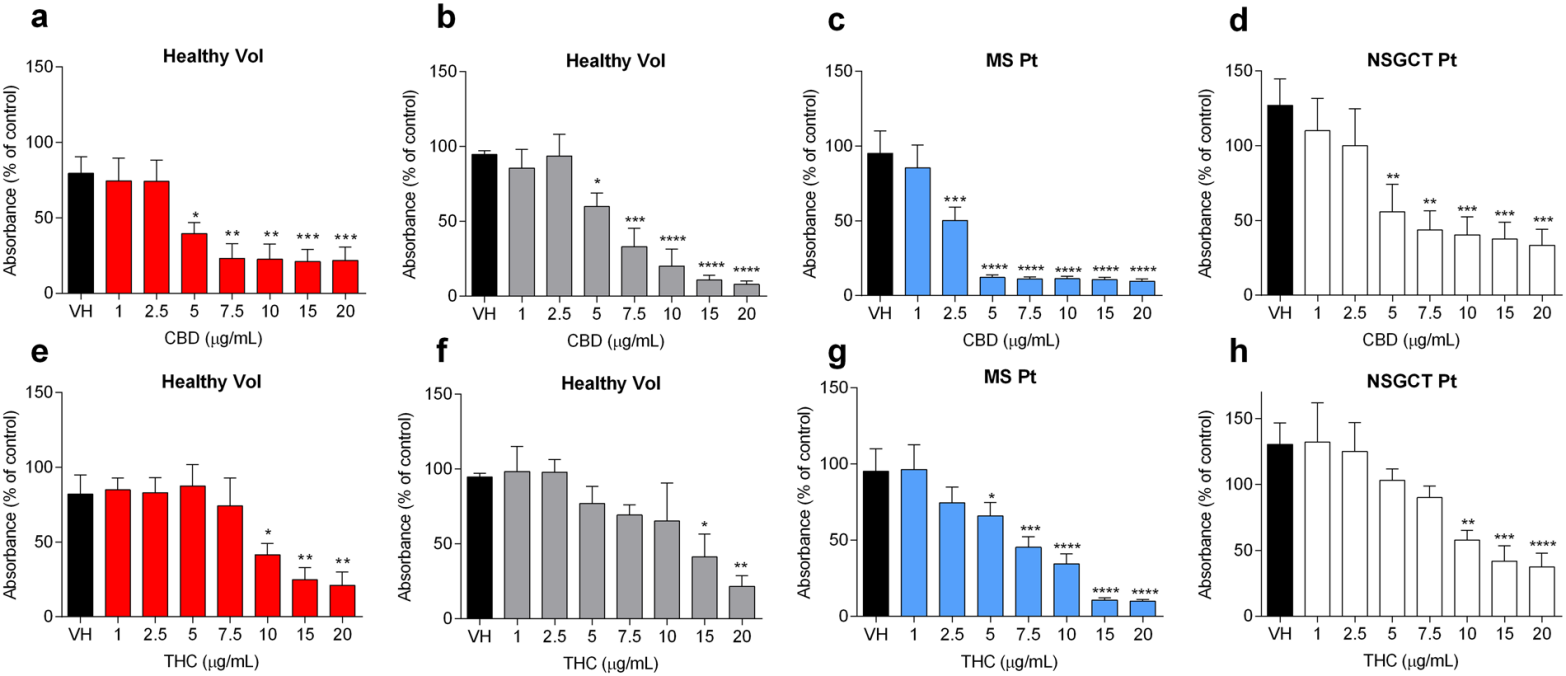
Effects of cannabinoids on the proliferation of peripheral blood mononuclear cells (PBMCs) isolated from human participants. Cells were stimulated by phytohaemagglutinin (PHA, 10 μg/mL) after incubation with CBD or THC at concentrations of 1–20 μg/mL. (**a**) Effect of DMSO-dissolved CBD on PBMCs isolated from healthy volunteers (*n* = 4). (**b**) Effect of CM-associated CBD on PBMCs isolated from healthy volunteers (*n* = 3). (**c**) Effect of DMSO-dissolved CBD on PBMCs isolated from multiple sclerosis (MS) patients (*n* = 7, as indicated in Table 1).(**d**) Effect of DMSO-dissolved CBD on PBMCs isolated from patients on chemotherapy to treat non-seminomatous germ cell tumours (NSGCT) (*n* = 7). (e) Effect of DMSO-dissolved THC on PBMCs isolated from healthy volunteers (*n* = 4). (**f**) Effect of CM-associated THC on PBMCs isolated from healthy volunteers (*n* = 3). (**g**) Effect of DMSO-dissolved THC on PBMCs isolated from MS patients (*n* = 7, as indicated in Table 1). (h) Effect of DMSO-dissolved THC on PBMCs isolated from patients on chemotherapy to treat NSGCT (*n* = 7). Values are expressed as percentage of absorbance compared to the control group of untreated cells, mean ± SEM. Samples run in triplicates. Statistical analysis was performed using one-way ANOVA with Fisher’s LSD test. Statistical differences compared to the vehicle-treated cells (VH); *P < 0.05; **P < 0.01; ***P < 0.001; ****P < 0.0001.

The immunomodulatory effects of CBD and THC were assessed on PBMCs isolated from human blood. Proliferation results showed that solutions of CBD and THC, as well as chylomicrons (CM)-associated CBD and THC can significantly inhibit the proliferation of PBMCs isolated from healthy volunteers at concentrations equal to or above 5 and 10 μg/mL, respectively (Fig. 5a,b,e,f). To assess the potential therapeutic value of targeting lipophilic cannabinoids to the intestinal lymphatic system, the immunosuppressive effect of cannabinoids on PBMCs isolated from patients suffering from autoimmune disease, particularly MS patients (Table 1) was evaluated. In this patient group, CBD markedly suppressed the proliferation of PBMCs at half of the concentrations observed for healthy volunteers PBMCs (Fig. 5c). Similar results were also found for THC (Fig. 5g).

**Table 1:** List of multiple sclerosis (MS) patients whose blood samples were used for the assessment of immunomodulatory effects of CBD and THC.

Patient code	Age (Y)	Gender	Type of MS	EDSS	Experiment
CMS-01	35	F	RRMS	1.5	Lymphocyte proliferation assay
CMS-02	55	F	RRMS	2.5	Lymphocyte proliferation assay
CMS-03	33	F	RRMS	1.5	Lymphocyte proliferation assay
CMS-04	51	F	RRMS	3	Lymphocyte proliferation assay
CMS-05	30	M	RRMS	3	Lymphocyte proliferation assay
CMS-06	55	F	RRMS	4	Lymphocyte proliferation assay
CMS-07	26	F	RRMS	2.5	Lymphocyte proliferation assay
CMS-08	69	F	SPMS*	6.5	Assessment of inflammatory cytokines
CMS-09	55	F	RRMS	2.5	Assessment of inflammatory cytokines
CMS-10	33	F	RRMS	2.5	Assessment of inflammatory cytokines
CMS-11	53	F	RRMS	4.5	Assessment of inflammatory cytokines
CMS-12	74	F	SPMS*	5.5	Assessment of inflammatory cytokines
CMS-13	30	F	RRMS	2	Assessment of inflammatory cytokines

RRMS, relapsing-remitting MS; secondary-progressive MS; EDSS, Expanded Disability Status Scale.

*Indicates secondary-progressive MS patients.

All patients were not on disease modifying drugs (newly diagnosed patients or at least one month after the discontinuation of disease modifying drugs).

All patients were known not to consume cannabinoids.

Furthermore, proliferation experiments were also conducted on PBMCs isolated from patients on chemotherapy regimens for treatment of non-seminomatous germ cell tumours (NSGCT, Table 2). In this set, CBD and THC showed anti-proliferative effects on PBMCs from NSGCT patients which were comparable to healthy volunteers, i.e. immunosuppressive effects at concentrations equal to and above 5 and 10 μg/mL, respectively (Fig. 5d,h).

**Table 2:** List of non-seminomatous germ cell tumour (NSGCT) patients whose blood samples were used for the assessment of immunomodulatory effect of CBD and THC.

Patient code	Age (y)	WBC (10^9^/L)	ANC (10^9^/L)	Ly (10^9^/L)
CTC-01	35	1.1	0.1	0.8
CTC-02	59	2.2	0.5	1.4
CTC-03	21	2	0.5	1.2
CTC-04	21	6.4	3.8	1.7
CTC-05	36	2.9	0.4	1.2
CTC-06	43	4.3	1.5	2.2
CTC-07	29	6.1	3.6	1.7
CTC-08	46	11	9.7	0.9
CTC-09	18	2.4	1.1	0.9
CTC-10	38	10.3	6.3	2.7

WBC, White Blood Cell Count; ANC, Absolute Neutrophil Count; Ly, Lymphocyte. Bloods were taken after a minimum of one cycle of chemotherapy at time of expected total white blood cell and neutrophil count recovery.

### Effects of CBD and THC on cytokine profiles of human lymphocytes

**Figure 6:**
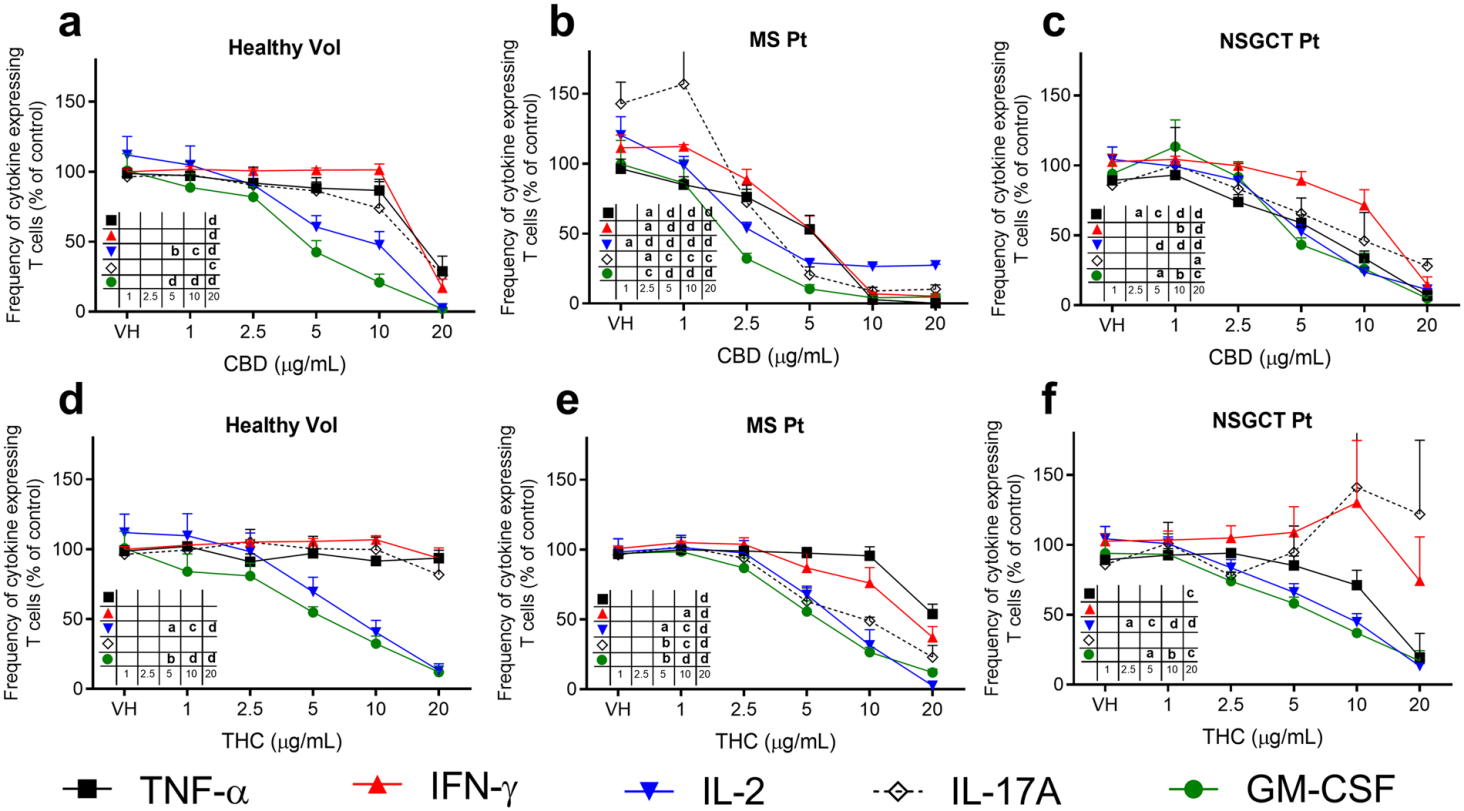
Effects of cannabinoids on the expression of inflammatory cytokines in CD3^+^ T cells isolated from human participants. Figure shows the frequency of TNF-α, IFN-γ, IL-2, IL-17A, and GM-CSF expressing CD3^+^ T cells isolated from human participants (% frequency of cytokines-expressing CD3^+^ T cells compared to the control group of untreated cells, mean ± SEM). Cells were stimulated by phorbol myristate acetate and ionomycin (PMA & I) in the presence of brefeldin A after incubation with cannabidiol (CBD) or ∆^9^-tetrahydrocannabinol (THC) at concentrations of 1–20 μg/mL. (**a**) Effect of CBD on PBMCs from healthy volunteers (*n* = 5). (**b**) Effect of CBD on PBMCs from multiple sclerosis (MS) patients (*n* = 4, as indicated in Table 1).(**c**) Effect of CBD on PBMCs from patients on chemotherapy to treat non-seminomatous germ cell tumours (NSGCT) (*n* = 3). (**d**) Effect of THC on PBMCs from healthy volunteers (*n* = 5). (**e**) Effect of THC on PBMCs from MS patients (*n* = 3, as indicated in Table 1). (**f**) Effect of THC on PBMCs from patients on chemotherapy to treat NSGCT (*n* = 3). Statistical analysis was performed using one-way ANOVA with Fisher’s LSD test. The inset table in the lower left corner of each panel refers to the statistical differences compared to the vehicle (DMSO)-treated cells (VH). a, *P* < 0.05; b, *P* < 0.01; c, *P* < 0.001; d, *P* < 0.0001.

Following *in vitro* activation of PBMCs, the intracellular expression of TNF-α, IFN-γ, IL-2, IL-17A, and granulocyte-macrophage colony-stimulating factor (GM-CSF) was assessed by means of flow cytometry. Similar to the proliferation assay, PBMCs were isolated from healthy volunteers, MS patients, and NSGCT patients. In PBMCs of healthy volunteers, CBD only significantly decreased TNF-α, IFN-γ, and IL-17A expressing T cells when incubated with cells at the highest tested concentration (20 μg/mL). IL-2 and GM-CSF expressing T cells, however, were suppressed at CBD concentrations at and above 5 μg/mL (Fig. 6a). In the case of THC, no appreciable effect was observed on TNF-α, IFN-γ, and IL-17A expressing T cells compared to the vehicle-treated cells. However, IL-2 and GM-CSF expressing T cells were inhibited at concentrations equal to and above 5 μg/mL (Fig. 6d). For PBMCs isolated from MS patients, the immunosuppressive effects of CBD and THC on the expression of the tested cytokines were significantly higher compared to the effects on cells isolated from healthy volunteers (Fig. 6b,e). Moreover, the effects of CBD and THC on the expression of the assessed cytokines from NSGCT patient T cells were comparable to healthy volunteers cells with some exceptions (Fig. 6). These include more prominent immunosuppressive effects of CBD on TNF-α and IFN-γ expressing T cells as well as the effects of THC on TNF-α and IL-2 expressing T cells from NSGCT patients (Fig. 6c,g). Representative flow cytometry histograms are shown in Supplementary Figure S1.

## Discussion

Highly lipophilic drugs such as the phytocannabinoids CBD and THC are good candidates for intestinal lymphatic transport^28^. In light of this, we have recently shown that the systemic bioavailability of CBD and THC can be significantly enhanced when administered orally in conditions facilitating intestinal lymphatic transport, specifically co-administration with dietary lipids^18^. The results of the current study indicate that the intestinal lymphatic transport of CBD and THC in rats was, indeed, enhanced following oral co-administration of lipids as denoted by the dramatic increase in the concentrations recovered in MLN (Fig. 1a,b). More importantly, the biodistribution of CBD and THC to lymphoid tissues in the intestinal lymphatic system (MLN) was substantially higher than the distribution to the largest lymphatic tissue in the central compartment, the spleen (Fig. 2a). It is also important to note the extremely high concentration of cannabinoids recovered in intestinal lymph fluid compared with plasma (Fig. 2b). Similar trends were previously reported for other lipophilic compounds, dexanabinol and PRS-211,220, when orally administered with LCT to rats^19^. Therefore, given our findings, we suggest that oral administration with dietary lipids has the potential to be an approach suitable for targeting delivery of CBD and THC to the intestinal lymphatic system. This targeting approach, as we have shown in our previous work, has the potential to increase the concentrations in plasma as well^18^.

The intestinal lymphatic system is the major host of immune cells. It has been proposed that the lymphatic system is an attractive target for immunomodulators whereby drugs can achieve high local concentrations and avoid systemic dilution^28,29^. This concept is supported by the fact that immune cells within the lymphatic system move more slowly and experience lower shear stress relative to those within the circulation^30^. In this study, the effect of CBD and THC on the proliferation of immune cells isolated from lymphoid tissues in the intestinal lymphatic system and spleen of rats was assessed. Both cannabinoids significantly inhibited the proliferation of mitogen-stimulated immune cells from MLN and spleen at concentrations that are highly unlikely to be achieved in rat plasma, but can be easily obtained in the intestinal lymphatic system after co-administration of lipids (Fig. 3). In addition, the results suggest that CBD has more potent anti-proliferative effect than THC (Fig. 3). Moreover, in this study, CBD and THC attenuated the expression of TNF-α and IFN-γ, key pro-inflammatory cytokines in the pathogenesis of autoimmune diseases^31^, by CD3^+^ T cells isolated from MLN and spleen (Fig. 4). In line with the anti-proliferative effect, cannabinoids also suppressed the frequency of inflammatory cytokines-expressing T cells at micromolar concentrations, which are achievable in the intestinal lymphatic system, but not in general circulation. To note, the reduced frequency of cytokines-expressing T cells can be potentially due to a decrease in the proliferation of cells. The immunosuppressive effects of CBD and THC on murine immune cells in the same concentration range were also reported elsewhere^12,32^. Intestinal lymphatic transport could be a potential mechanism of previously reported therapeutic effects of CBD and THC in murine models of autoimmune diseases in which the cannabinoids were administered orally with substantial amounts of lipids^3,10^.

To assess the immunomodulatory effect of cannabinoids on human cells, proliferation assays were performed on PBMCs isolated from venous blood of human volunteers. Our results demonstrate that CBD and THC, at relatively high concentrations, inhibit the proliferation of mitogen-stimulated PBMCs isolated from healthy volunteers (Fig. 5a,e). These results are consistent with previous reports^13^. Importantly, these concentrations would not be achievable in systemic blood circulation even if high oral doses of cannabinoids were consumed. Consroe *et al*.^33^ reported that the maximum plasma concentration recovered following repeated oral administration of 10 mg/kg/day CBD (in a small-amount lipid-based formulation) in humans was 25 ng/mL. This is considerably lower than the 5 μg/mL concentration required to significantly inhibit PBMCs proliferation in this study (Fig. 5a). In contrast, high doses of THC are limited by the psychotropic side effects reported for this drug^34^. Nevertheless, given the results obtained in this study, it is likely that oral administration of CBD and THC in humans in conditions facilitating intestinal lymphatic transport (with lipid dose in the range of 10 g^35^) can result in significant increases in intestinal lymphatic transport similar to what has been obtained for rats in this study. This is also supported by the fact that CBD and THC showed comparable association values with human and rat CM^18^. It should be noted that CM association in the enterocytes is a prerequisite for the intestinal lymphatic delivery of lipophilic compounds when orally administered with lipids^18,19,36,37^. Importantly, in the current study, CM-associated cannabinoids were still able to inhibit the proliferation of PBMCs isolated from healthy volunteers, showing similar effects to cannabinoids in solution (Fig. 5b,f). Therefore, our results suggest that the fact that cannabinoids are delivered to the intestinal lymphatic system in a CM-associated form would not reduce the immunomodulatory effects. A possible explanation for this maintained effect in CM-associated form is that lymphocytes possess lipoprotein lipase enzyme activity, which enables them to utilise fatty acids and triglycerides from CM^38^ and therefore to be exposed to cannabinoids.

In addition, our results indicate that PBMCs isolated from MS patients are more sensitive to the immunomodulatory effects of cannabinoids compared to PBMCs isolated from healthy volunteers (Fig. 5c,g). This could be related to the up-regulation of cannabinoid CB_2_ receptors in the immune cells of MS patients^39^. These receptors are thought to be involved in the immunosuppressive effect of cannabinoids^32,40^. Interestingly, some other reports suggested that cannabinoids exert their immunosuppressive effect by CB_2_-independent mechanisms as well^41^. Another aspect demonstrated in this study is that cannabinoids have comparable anti-proliferative effects on PMBC isolated either from blood of cancer patients under chemotherapy regimens or from healthy volunteers (Fig. 5d,h). However, cancer patients on chemotherapy usually have low or low-borderline blood lymphocyte counts^42^, which was also the case in this study (Table 2). Therefore, there could be a potential effect of further immunosuppression in some people, such as cancer patients, when cannabinoids are administered in conditions that facilitate intestinal lymphatic transport.

It is widely accepted that TNF-α, IFN-γ, and IL-2 produced by T_H_1 cells are actively involved in the pathogenesis of many autoimmune diseases^43^. Recently, T_H_17 cells (which produce IL-17A) have emerged as a major factor in the pathogenesis of autoimmune diseases, as well as the contribution of GM-CSF to drive the inflammatory effects of T_H_17^44^ and T_H_1^45,46^ cells. In this study, CBD and THC induced a profound decrease in the frequency of IL-2 and GM-CSF expressing T cells separated from healthy volunteers. This is consistent with the demonstration of a link between IL-2 production and GM-CSF induction^47^. CBD showed higher immunosuppressive effect than THC as manifested by the effect on TNF-α, IFN-γ, and IL-17A expressing T cells (Fig. 6a,d). For PBMCs isolated from MS patients, similar to the anti-proliferative effect, cannabinoids displayed more potent suppression of cytokine expression compared to cells from healthy volunteers (Fig. 6b,e). Yet, these effects were only observed at micromolar concentrations, consistent with previous reports^13,48^. Collectively, the effects of cannabinoids on lymphocyte proliferation and the frequency of cytokines-producing T cells explored in the current study suggest that targeting lipophilic cannabinoids to the intestinal lymphatic system for enhanced immunomodulatory effects in the treatment of autoimmune diseases could be a promising therapeutic approach. This approach could extend the therapeutic value of cannabinoids currently being used for symptomatic relief in MS patients^25^ to a disease modifying treatment, which could delay the progression of MS. Moreover, the results suggest that CBD has higher therapeutic effectiveness in autoimmune diseases compared with THC as it has more pronounced immunomodulatory effects, is devoid of psychotropic side effects, and is well tolerated in humans following acute and chronic intake of relatively high doses^49^.

On the other hand, adequate levels of the above-mentioned cytokines are important to maintain adaptive immune responses to fight infections^50,51^. In this study, a substantial decrease in the frequency of cytokines-expressing T cells has been demonstrated with cannabinoids in PBMCs isolated from cancer patients under chemotherapy regimen (Fig. 6c,f). This can potentially further deteriorate chemotherapy-induced immunosuppression in these patients. In addition, it has been reported that CBD and THC have inhibitory immunomodulatory effects on innate immune cells, particularly macrophages and natural killer cells (NK)^52^. It is well recognised that some cancer patients self-medicate and consume cannabis or cannabis-based medicinal formulations orally to alleviate nausea and vomiting associated with chemotherapy^26^. Given the results of this study, in this patients group, oral administration of immunosuppressive drugs such as cannabinoids in conditions facilitating intestinal lymphatic transport requires caution.

In summary, it has been demonstrated in this work that oral co-administration of cannabis or cannabis-based medicines with lipids results in extremely high levels of lipophilic cannabinoids in the intestinal lymphatic system and prominent immunomodulatory effects. Therefore, administering cannabinoids with a high-fat meal, as cannabis-containing food, or in lipid-based formulations has the potential to be a therapeutic approach to improve the treatment of MS, or indeed other autoimmune disorders. Whether cannabinoids as used in this study can also induce regulatory cytokines in addition to suppressing inflammatory ones as shown here remains to be determined. Further studies would be required to assess if both lymph and peripheral concentrations of cannabinoids have to be high for effective immunomodulation or only targeting to lymphatic system is sufficient. In addition, more work would have to be done to elucidate if cannabinoids could be used alone or as an adjuvant to other treatment. However, in immunocompromised patients, administration of cannabinoids in this way could potentially deepen the immunosuppressive effects. Further studies will be needed to evaluate the clinical significance of these effects.

## Methods

### Cannabinoids

CBD and THC were donated by GW Research Ltd (Cambridge, UK).

### Animals

All experiments and procedures were approved by the UK Home Office in accordance with the Animals [Scientific Procedures] Act 1986. Experiments were performed using male Sprague-Dawley rats (Charles River Laboratories) weighing 300–349 g. The rats were housed in the University of Nottingham Bio Support Unit, and kept in a temperature-controlled and 12 hours light-dark cycle environment with free access to water and food. All experiments were performed in accordance with the approved guidelines.

### Human samples

The protocol for the preparation of human plasma-derived CM emulsion was approved by the Faculty of Medicine and Health Sciences Research Ethics Committee, Queens Medical Centre, Nottingham University Hospitals, Nottingham, UK (BT12102015 CBS SoP). Lymphocyte proliferation and flow cytometry experiments conducted on PBMCs isolated from healthy volunteers and MS patients (Table 1) were approved by the Research Ethics Committee East Midlands – Nottingham 2, Nottingham, UK (08/H0408/167/AM05). Lymphocyte proliferation and flow cytometry experiments conducted on PBMCs isolated from NSGCT patients (Table 2) were approved by Nottingham Health Sciences Biobank at Nottingham University Hospitals, Nottingham, UK (ACP162). Informed written consent was obtained from all participants. The methods were carried out in accordance with the approved guidelines.

### Biodistribution of cannabinoids to MLN of rats

Following 5 days of acclimatisation, rats were fasted overnight with free access to water. Animals were orally administered CBD and THC in lipid-free (12 mg/mL solution in propylene glycol–ethanol–sterile water (80:10:10, v/v/v)) and LCT-based formulation (12 mg/mL solution in sesame oil) at a dose of 12 mg/kg. Rats were euthanised at the pre-determined time points of *t*_max_ and *t*_max_ − 1 h, time points are listed in Supplementary Table S1. MLN were then collected as previously described^53^. Briefly, animal carcass was laid on back and the ventral abdominal wall was incised to expose the intestine. MLN were removed from the mesenteric tissue in the abdominal cavity and placed in an Eppendorf tube. MLN were gently dissected from surrounding tissue, weighed, and homogenized with saline (1:3 w/v) on ice at 18,000 rpm for 3 min (POLYTRON^®^ PT 10–35 GT, Kinematica AG, Luzern, Switzerland). Homogenates were then assessed for CBD and THC content as described in the analytical method section.

### Biodistribution of cannabinoids to plasma, intestinal lymph fluid, and spleen of rats

Following 5 days of acclimatisation, animals were fasted overnight with free access to water. CBD and THC were orally administered to rats in an LCT-based formulation (12 mg/kg). Animals were euthanized two hours following oral administrations. Blood samples were collected from the posterior vena cava. Lymph samples were collected from the mesenteric lymph duct. Briefly, the duct was ligated using 3–0 silk suture. A 25 G needle connected to 1 mL syringe was used to collect lymph form the duct (~50 µL of lymph was collected form each animal). In addition, spleen was collected, weighed, and homogenized as described above for MLN. Cannabinoids concentrations were then determined in plasma, intestinal lymph fluid, and spleen homogenates as described in the analytical method section.

### Preparation of single-cell suspension from MLN and spleen of rats

Following 5 days of acclimatisation, animals were euthanized and the ventral abdominal wall was incised to expose the intestine. MLN and spleen were aseptically collected. MLN were gently dissected from surrounding tissue and spleen was scored with a clean scalpel before being mashed on cell strainer (70 μm Nylon, Corning Falcon™). Red blood cells in the cell suspension of the splenocytes were lysed by lysing buffer (BD Bioscience). Immune cells from MLN and splenocytes were then washed twice with PBS. Cell suspension was centrifuged (400 *g*, 5 min at room temperature) and resuspended in complete RMPI-1640 culture medium (RMPI-1640 culture medium with L-glutamine supplemented with 10% fetal bovine serum (FBS) and 1% penicillin-streptomycin, all purchased from Sigma Aldrich) at concentration of 1.2 × 10^6^ cells/mL to be used for proliferation and flow cytometry experiments.

### Isolation of PBMCs from human blood

PBMCs were obtained from heparinised venous blood of healthy adult volunteers, MS patients (eleven relapsing-remitting and two secondary-progressive MS patients, Table 1), and NSGCT patients (Table 2) by density centrifugation (800 *g*, 30 min, 20 °C) using Histopaque^®^−1077 (Sigma Aldrich). Cells were suspended in complete RMPI-1640 culture medium at concentrations of 7.5 × 10^5^ and 1 × 10^6^ cells/mL for lymphocyte proliferation assay and flow cytometry experiments, respectively.

### Preparation of human CM-associated CBD and THC

Human plasma-derived CM emulsion was prepared from three male healthy human volunteers as previously described^18^. The uptake of CBD and THC by human CM emulsion was performed as previously described with small modifications^36^. Briefly, stock solutions of CBD and THC (12 mg/mL) were prepared in propylene glycol–ethanol (90:10, v/v). A volume of 25 µL of cannabinoid stock solution was added to 2 mL of the CM emulsion and incubated at 37 °C for 1 hour with continuous mixing. Following incubation, the density of the emulsion was adjusted to 1.1 g/mL using potassium bromide (Sigma Aldrich). CM were then separated by density gradient ultracentrifugation (SORVALL^®^ TH-641 Rotor, Thermo Fisher Scientific, 268,350 *g*, 35 min, 15 °C). The top 1 mL layer was collected following ultracentrifugation using a glass pipette. The concentration of CBD and THC in CM emulsion was assessed as described in the analytical methods section below. CM-associated cannabinoids were kept at 4 °C pending proliferation assay experiments (<24 hours).

### Lymphocyte proliferation assay

Immune cells from rats (MLN and spleen cells) and PBMCs from human participants were cultured in flat clear-bottom 96-well microplates (Thermo Scientific Nunc^®^) at concentration of 8.4 × 10^4^ and 5.2 × 10^4^ cells/well, respectively. Working stock solutions of CBD and THC in RMPI-1640 culture medium-DMSO (99:1, v/v) were prepared at concentrations of 10, 25, 50, 75, 100, 150, and 200 μg/mL. Working stock solutions of CM-associated cannabinoids were also prepared at the same aforementioned concentrations. Cannabinoids were incubated with cells at final concentrations of 1, 2.5, 5, 7.5, 10, 15, and 20 μg/mL in a humidified atmosphere of 5% CO_2_ at 37 °C for 30 min. Cells were then stimulated by the T cell-selective mitogen Phytohaemagglutinin^54^ (PHA, 10 μg/mL, Sigma Aldrich) and incubated in a humidified atmosphere of 5% CO_2_ at 37 °C for 2 days. Cell proliferation was assessed by enzyme-linked immunosorbent assay (ELISA) based on bromo-2′-deoxyuridine (BrdU) incorporation into newly synthetised DNA according to the manufacturer protocol (Roche Applied Science, Roche Diagnostics Ltd, UK). Finally, the absorbance of these wells was observed at 370 nm, with reference wavelength at 492 nm using plate reader (EnVision^®^ Multilabel Plate Reader, PerkinElmer Inc., USA). Absorbance values were normalised to the absorbance of culture medium-treated cells.

### Flow cytometry analysis

Freshly isolated immune cells of MLN and splenocytes from rats and thawed PBMCs from human participants were incubated with CBD or THC (1–20 μg/mL) for 30 min in FACS tubes. Cells were then stimulated with phorbol myristate acetate and ionomycin (PMA & I) in the presence of brefeldin A and incubated in a humidified atmosphere of 5% CO_2_ at 37 °C for 5 hours. After stimulation, cells were washed with PBS and centrifuged to pellet (290 *g*, 5 min, 20 °C). Cell pellet was resuspended and labelled with Zombie UV™ Fixable Viability kit according to the manufacturer’s protocol (Biolegend) for the purpose of excluding dead cells during the analysis of data (the effect of cannabinoids on the variability of immune cells isolated from healthy volunteers is presented in Supplementary Figure S2). Fixation and permeabilization was performed using BD Cytofix/Cytoperm™ kit according to the manufacturer’s protocol (BD Bioscience). Rat’s immune cells were labelled with APC anti-rat CD3, PE anti-mouse / rat TNF-α, and FITC anti-rat IFN-γ antibodies (Biolegend). Human’s PBMCs were labelled with BV421 anti-human TNF-α and PerCP/Cy5.5 anti-human IL-2 antibodies (Biolegend), ECD anti-human CD3, FITC anti-human IFN-γ antibodies (Beckman Coulter), and PE anti-human IL-17A, and APC anti-human GM-CSF antibodies (eBioscience). Isotype and fluorescence minus one (FMO) controls were prepared for all antibodies in each flow cytometry run. Data were collected on MoFlo^®^ Astrios™ EQ flow cytometer and analysed using Kaluza analysis software v 1.5 (Beckman Coulter). The gating strategy used for data analysis is illustrated in Supplementary Figure S3.

### Analytical methods

The concentrations of CBD in rat plasma, intestinal lymph fluid, MLN homogenates, spleen homogenates, and human CM samples, as well as THC concentrations in rat plasma, intestinal lymph fluid, and human CM samples were determined using HPLC system (Waters Alliance 2695 separations module) equipped with photodiode array ultraviolet (UV) detector (Waters 996)^18,55^. Concentrations of THC in MLN and spleen homogenates were determined by LC-MS/MS system consisted of Quattro Ultima triple-quadrupole mass spectrometer (Waters) coupled with Agilent HPLC system (1100 Series, Agilent Technologies) as previously described for the detection of THC in microsomal samples^56^. Chromatographic conditions for the detection of CBD and THC in rat plasma, intestinal lymph fluid, MLN homogenates, spleen homogenates, and human CM samples are summarized in Supplementary Table S2.

### Statistical analysis

Results are expressed as mean ± standard error of the mean (SEM). Statistical differences between data sets were assessed using either ANOVA with Fisher’s LSD test or unpaired two-tailed Student’s *t*-test, as appropriate. A *P* value < 0.05 was considered to represent a significant difference.

## Electronic supplementary material

Supplementary Material
