# Supplementary material for: Oral administration of cannabis with lipids leads to high levels of cannabinoids in the intestinal lymphatic system and prominent immunomodulation
Source: Sci Rep. 2017 Nov 6;7:14542. doi: 10.1038/s41598-017-15026-z (PMC5674070; doi:10.1038/s41598-017-15026-z)
Supplement: Supplementary file 1 — Supplementary Material [file 41598_2017_15026_MOESM1_ESM.pdf]

Oral administration of cannabis with lipids leads to high levels of cannabinoids in the intestinal lymphatic system and prominent immunomodulation

Atheer Zgair[1,2], Jong Bong Lee[1], Jonathan C. M. Wong[1], Dhiaa A. Taha[1], Jehan Aram[3], Daisy Di Virgilio[1], Joshua W. McArthur[1], Yu-Kit Cheng[1], Ivo M. Hennig[4], David A. Barrett[1], Peter M. Fischer[1], Cris S. Constantinescu[3], and Pavel Gershkovich[1,*]

[1]School of Pharmacy, University of Nottingham, Nottingham NG7 2RD, United Kingdom

[2]College of Pharmacy, University of Anbar, Anbar, Iraq

[3]Division of Clinical Neuroscience, University of Nottingham and Queen's Medical Centre, Nottingham NG7 2UH, United Kingdom

[4]Nottingham City Hospital, Nottingham University Hospitals NHS Trust, Nottingham NG5 1PB, United Kingdom

*To whom correspondence should be addressed:

Pavel Gershkovich, PhD

Division of Molecular Therapeutics and Formulation

School of Pharmacy

Centre for Biomolecular Sciences

The University of Nottingham

University Park, NG7 2RD

Nottingham, UK

Tel: +44 (0) 115 846 8014

Fax: +44 (0) 115 951 3412

Email: Pavel.Gershkovich@nottingham.ac.uk

Supplementary Information

Table S1. Times of maximum concentration in plasma (t_{max}) and one-hour prior to t_{max} (t_{max} – 1h) following oral administration of lipid-free formulation (12 mg/kg) and long-chain triglyceride (LCT)-based formulation (12 mg/kg) of cannabidiol (CBD) and Δ^9-tetrahydrocannabinol (THC) to rats[18].

Cannabinoid	Formulation	t_{max} (h)	t_{max} – 1h
CBD	lipid-free	3	2
	LCT-based	3	2
THC	lipid-free	2	1
	LCT-based	3	2

Table S2. Chromatographic conditions for the detection of cannabidiol (CBD) and Δ^9-tetrahydrocannabinol (THC) in rat plasma, intestinal lymph fluid, mesenteric lymph nodes (MLN), spleen, and human chylomicron (CM) samples[18,53,54].

	Medium	Mobile phase	Stationary phase	Flow rate (mL.min^{-1})	Oven temperature (°C)	IS	Detector/ conditions
CBD	Plasma and lymph	ACN and Water (62:38, v/v)	ACE C18-PFP 150 × 4.6 mm, 3 µm	1	55	DDT	UV/ 220 nm
	MLN	ACN and Water (75:25, v/v)	ACE Excel Super C18 100 × 4.6 mm, 5 µm	0.8	43	DDT	UV/ 230 nm
	Spleen	ACN and Water (75:25, v/v)	ACE Excel Super C18 100 × 4.6 mm, 5 µm	0.8	43	DDT	UV/ 230 nm
	Human-CM	ACN and Water (75:25, v/v)	ACE Excel Super C18 100 × 4.6 mm, 5 µm	0.8	43	DDT	UV/ 210 nm
THC	Plasma and lymph	ACN and Water (62:38, v/v)	ACE C18-PFP 150 × 4.6 mm, 3 µm	1	55	DDT	UV/ 220 nm
	MLN	0.1% (v/v) formic acid in ACN and Water (90:10, v/v)	Waters XBridge C18 75 × 2.1 mm, 2.5 µm	0.3	60	$VitD_3$	MS/MS THC: + 315.2/193.0 $VitD_3$: + 385.3/259.3
	Spleen	0.1% (v/v) formic acid in ACN and Water (90:10, v/v)	Waters XBridge C18 75 × 2.1 mm, 2.5 µm	0.3	60	$VitD_3$	MS/MS THC: + 315.2/193.0 $VitD_3$: + 385.3/259.3
	Human-CM	ACN and Water (90:10, v/v)	ACE Excel Super C18 100 × 4.6 mm, 5 µm	0.6	43	PB	UV/ 220 nm

IS, internal standard; DDT, 4,4-dichlorodiphenyltrichloroethane; PB, probucol.

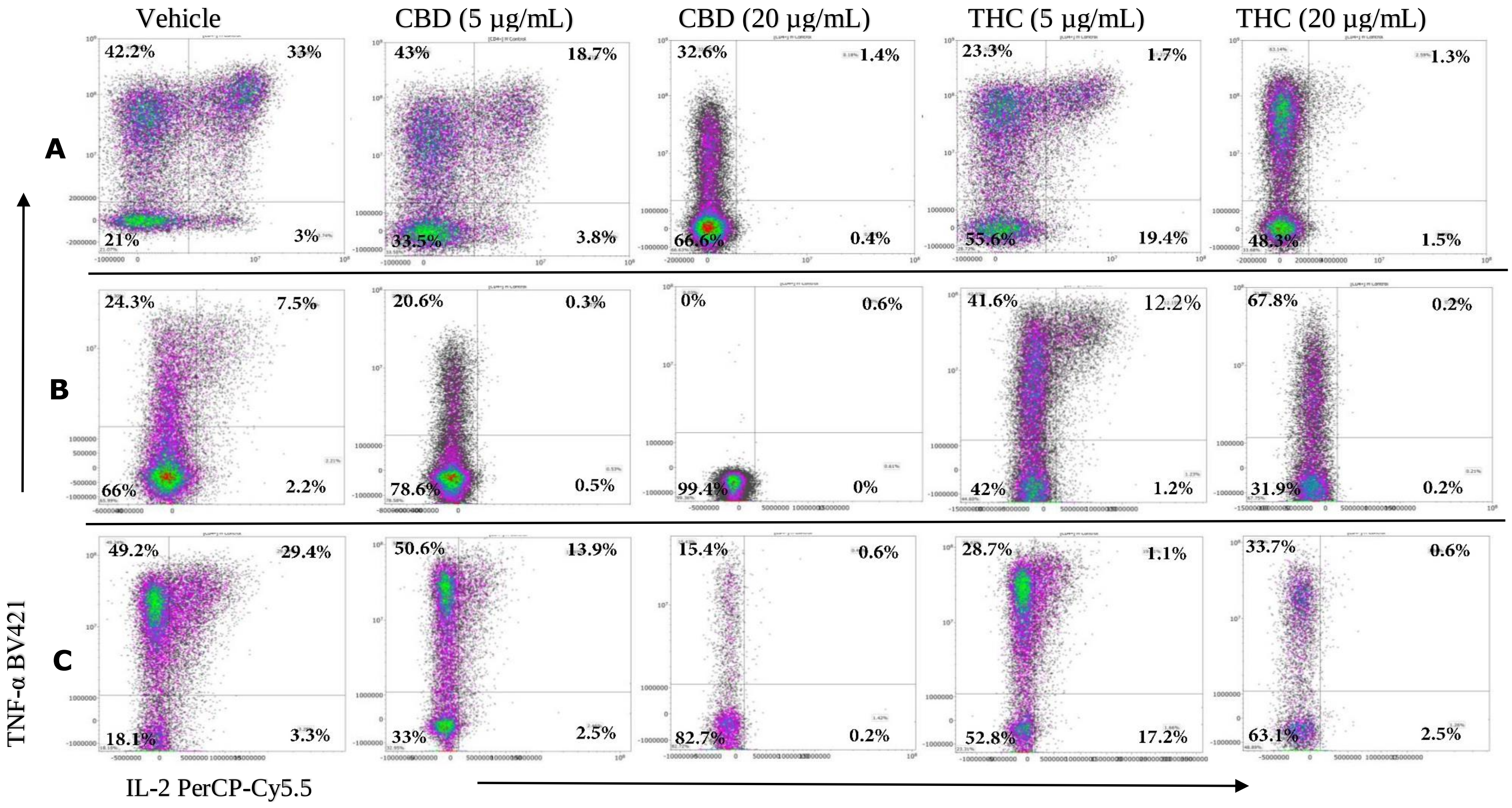

Figure S1. Representative flow cytometry histograms showing the effects of cannabidiol (CBD) and Δ^9-tetrahydrocannabinol (THC) at concentrations of 5 and 20 µg/mL on TNF-α and IL-2 expressing $CD3^+$ T cells isolated from human participants. Cells were stimulated by phorbol myristate acetate and ionomycin (PMA & I) in the presence of brefeldin A. **Panel A:** Effect of CBD and THC on PBMC from a healthy volunteer, **Panel B:** Effect of CBD and THC on PBMC from a multiple sclerosis (MS) patient, **Panel C:** Effect of CBD and THC on PBMC from a patient on chemotherapy to treat non-seminomatous germ cell tumours (NSGCT).

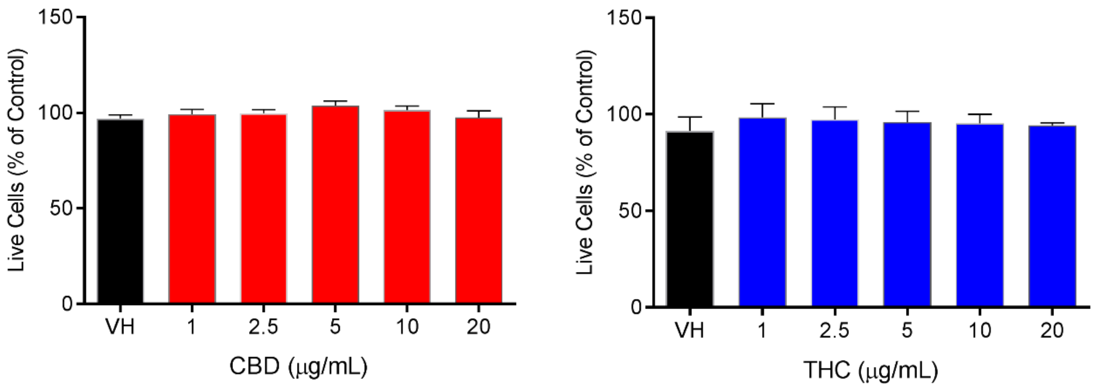

Figure S2. Effects of cannabidiol (CBD) and Δ^9-tetrahydrocannabinol (THC) at concentrations of 1-20 µg/mL on the viability of $CD3^+$ T cells isolated from healthy human participants (n = 5). Statistical analysis was performed using one-way ANOVA with Fisher's LSD test. No statistical differences were observed compared to the vehicle (DMSO)-treated cells (VH).

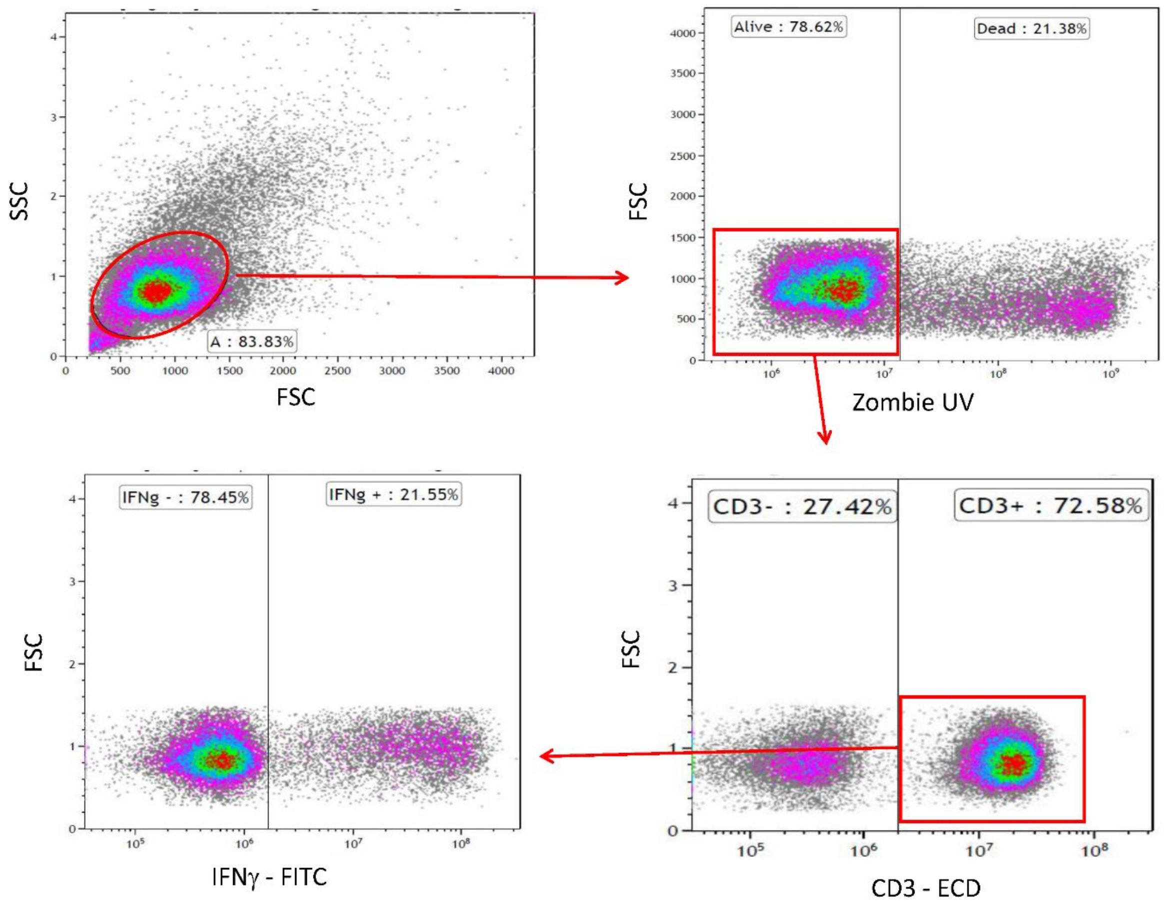

Figure S3. The gating strategy used for the analysis of flow cytometry data for the assessment of intracellular cytokines (IFNγ as an example) as a response to treating immune cells with cannabidiol (CBD) and Δ^9-tetrahydrocannabinol (THC).
